# Rapid and Nondestructive Detection of Proline in Serum Using Near-Infrared Spectroscopy and Partial Least Squares

**DOI:** 10.1155/2022/4610140

**Published:** 2022-10-19

**Authors:** Kejing Zhu, Shengsheng Zhang, Keyu Yue, Yaming Zuo, Yulin Niu, Qing Wu, Wei Pan

**Affiliations:** ^1^Organ Transplantation Department, The Affiliated Hospital of Guizhou Medical University, 28 Guiyi Rd, Guiyang 550004, Guizhou, China; ^2^Innovation Laboratory, The Third Experiment Middle School, Guizhou Key Laboratory for Information System of Mountainous Areas and Protection of Ecological Environment, Guizhou Normal University, 116 Baoshan North Rd, Guiyang 550001, Guizhou, China; ^3^Institute of Rail Transit, Tongji University, 4800 Caoan Highway, Shanghai 201804, China; ^4^School of Basic Medical Sciences, Hubei Key Laboratory of Wudang Local Chinese Medicine Research, Hubei University of Medicine, 30 Renmin South Rd, Shiyan 442000, Hubei, China; ^5^Guizhou Prenatal Diagnosis Center, The Affiliated Hospital of Guizhou Medical University, 28 Guiyi Rd, Guiyang 550004, Guizhou, China

## Abstract

Proline is an important amino acid that widely affects life activities. It plays an important role in the occurrence and development of diseases. It is of great significance to monitor the metabolism of the machine. With the great advantages of deep learning in feature extraction, near-infrared analysis technology has great potential and has been widely used in various fields. This study explored the potential application of near-infrared spectroscopy in the detection of serum proline. We collected blood samples from clinical sources, separated the serum, established a quantitative model, and determined the changes in proline. Four algorithms of SMLR, PLS, iPLS, and SA were used to model proline in serum. The root mean square errors of prediction were 0.00111, 0.00150, 0.000770, and 0.000449, and the correlation coefficients (Rp) were 0.84, 0.67, 0.91, and 0.97, respectively. The experimental results show that the model is relatively robust and has certain guiding significance for the clinical monitoring of proline. This method is expected to replace the current mainstream but time-consuming HPLC, or it can be applied to rapid online monitoring at the bedside.

## 1. Introduction

Amino acids are fundamental units of life, and the existence of amino acids has also been detected in extraterrestrial sources (such as meteorites). A crucial step in the chemical evolution of life is the dehydration and condensation of amino acids into peptides, which are the source of all life [1, 2]. Polypeptides are required to fold into proteins in order to perform biochemical functions. Amino acids have some function other than their canonical function related to protein synthesis. For example, amino can drive cell proliferation and cell growth. Glutamate can act as a signaling molecule that regulatescell function, especially for immune cell function. Most amino acids can provide nutritional functions. All in all, amino acids are essential for humanlife activities.. Mainly, there are 20 amino acids inside the human body [3–5]. According to nitrogen balance experiments, there are 8 kinds of amino acids that cannot be synthesized autonomously, called essential amino acids, and the remaining 12 kinds of amino acids, which do not need to be supplied by food, are called nonessential amino acids. In addition to their role as a nutrition supporter, amino acids have additional specific regulatory function such as proline.

Proline (Pro) is synthesized from glutamic acid (Glu), the intermediate is *γ*-glutarate-semialdehyde (GSA), which spontaneously cyclizes to Δ-pyrroline-5-carboxylate (P5C) [6, 7], and then, P5C is reduced to proline by P5C reductase (P5CR) [8, 9]. Meanwhile, proline can be converted to glutamate via proline catabolism regulated by proline dehydrogenase 1 (PRODH) and P5C dehydrogenase (P5CDH), and in this process, the electron transport chain (ETC) [10] in the redox of proline will increase the production of ROS in mitochondria. Besides their harmful effect, ROS also have signaling function, which may be an important basis for proline to affect a wide range of cellular processes. Numerous studies have shown that Pro influences broad biological processes, including bioenergetics, differentiation, growth, lifespan, and apoptosis. In addition, proline is also closely related to oxidative stress [11–19].

According to the above findings, Pro is maintained at a relatively constant level and renewed rapidly in our body, which plays an important role in the initiation and development of diseases. Thus, monitoring the changes of substance concentration and metabolism under physiological and pathophysiological conditions can contribute to the treatment and prevention method of the disease. At present, amino acids in blood determination methods include high-performance liquid chromatography, amino acid analysis instrument method, liquid chromatography-mass spectrometry, capillary electrophoresis, and gas chromatography [20]. In clinical practice, high-performance liquid chromatography, base acid analysis instrument method, and liquid chromatography-mass spectrometry are used to detect the content of amino acids in patients [21]. However, most amino acids do not contain chromophores and cannot be directly detected by commonly used ultraviolet and fluorescence detectors [22]. It is usually necessary to perform complex precolumn or postcolumn derivatization of the target components to improve the sensitivity and separation selection characteristics of instrument analysis. An amino acid analyzer is considered the gold standard for quantitative analysis of amino acid in biological samples [23]. Nevertheless, its main drawbacks are the long test waiting time for the result and large sample amount. In addition, the technical threshold of amino acid analyzers is relatively high, mostly dependent on imports, and the price is quite expensive. Nemkov et al. reported an improved ultrahigh-performance liquid chromatography (UHPLC)-mass spectrometry (MS) method, which runs for 3 minutes to detect a variety of amino acids in samples [24]. The improved method exhibits several advantages, such as a fast, sensitive, and accurate response, which can be used as a new metabolic diagnostic laboratory approach alternative to amino acid analyzers. However, this UHPLC-MS method has some disadvantages. For example, the chromatographic part has higher requirements for preprocessing conditions and the mobile phase and mass spectrometers are expensive. UPLC-MS has strict requirements for analysis conditions and high analysis costs, which limit amino acid monitoring from further developing and becoming more widespread. Anyway, the conventional ways are expensive, complex to operate, require professional personnel, and have long detection times. Consequently, we need a detection method that is easy to handle, fast in analysis, low in detection cost, and capable of being widely popularized, which is the current development direction of amino acid analysis.

It has been reported that the use of near-infrared technology to detect amino acids in biological samples [25–30] has a certain prompting effect on our research. The near-infrared (NIR) [31] spectral range is 780–2526 nm. Over frequency vibration or rotation of chemical bonds contained in organic substances can obtain the absorption spectrum of the sample in the infrared region by means of transmission or diffuse reflection, which can be used to predict the unknown chemical composition of samples. Among them, quantitative analysis based on near-infrared technology is gradually recognized by more and more people, and relevant national and local industry standards are gradually increasing, such as GB/T36691-2018, NY/T2794-2015, and other national standards. These national standards all use infrared technology to detect the content of relevant components in substances. In addition, it has also received attention in the fields of near-infrared quantitative analysis, military industry, quality inspection, etc., and related models have been developed. The Xi'an Institute of Modern Chemistry established a rapid determination of *α*-HMX impurity crystal form in octogen (HMX) explosives by using near-infrared spectroscopy [32]. In terms of the content method, the Zhongshan Entry-Exit Inspection and Quarantine Bureau has established dozens of quick-test textile quantitative analysis models such as cotton/polyester, cotton/spandex, and viscose/polyester [33]. After repeated verification and improvement, they have been used in multiple exits. In the daily inspection work of the Inspection and Quarantine Bureau, Li Jingjing and others used online near-infrared spectroscopy to monitor the polysaccharide content, soluble solid content, and PH value of Chinese herbal medicine oral liquid, which enhances the controllability of the production process and helps improve different batches of product quality consistency [34]. However, the main substrate of blood is water. Due to the structural characteristics of water molecules, their near-infrared spectrum is susceptible to “disturbance” factors (temperature, concentration, solute changes, etc.). When the environment of the water molecule changes (equivalent to adding other components to the water), the spectrum of the water molecule will also change accordingly. In other words, the near-infrared spectrum of water contains a lot of information about solutes. Shao Xueguang carried out a series of research work in this methodological field. With the changes in “disturbance factors,” a quantitative and qualitative analysis method of the solution system was established [35], which is an important theoretical basis for the detection of proline in blood in this study. It is precisely because of the advantages of near-infrared spectroscopy technology, such as simple operation, fast analysis speed, low cost of detection, and nondestructive samples, making near-infrared spectroscopy an ideal alternative method for proline detection.

Overall, proline metabolism plays an important role in the process of occurrence and development of diseases. Thus, monitoring the changes in the concentration of proline under physiological or pathological conditions is significant for disease prevention and management in the future. In this research, we established and optimized the proline detection method based on the built-in TQ-analysis algorithm, stepwise multiple linear regression algorithm, partial least squares algorithm, interval partial least squares algorithm, and simulated annealing, to achieve rapid and nondestructive detection of serum proline.

## 2. Materials and Methods

### 2.1. Materials and Instrumentation

The serum samples were obtained from the Affiliated Hospital of Guizhou Medical University (Guiyang, China), and the proline content was determined by HPLC. This study was approved by the Human Research Ethics Committee at the Affiliated Hospital of Guizhou Medical University. Fourier-transform near-infrared spectra analyzer (Antaris II, Thermo Fisher, Waltham, MA, USA), transmission module sampling system, RESULT-Integration workflow design software, TQ Analyst 9 (Thermo Fisher Scientific, Waltham, MA, USA), Omnic software (OMNIC 8.2, Thermo Nicolet Corporation, Waltham, MA, USA) and MATLAB2019 were used for kinetic studies.

### 2.2. Sample Collection and Processing

Blood was collected by the clinical nurse, and the fast serum tube was selected for temporary storage and transferred to the laboratory at 4° cold chain. After confirming that there was no hemolysis, after confirming the receipt, we let it stand at room temperature for 30 minutes. It was centrifuged at 3500 r/min for 5 minutes to separate the serum and stored at −80°.

### 2.3. Workflow Establishment and Serum Spectrum Collection

We opened RESULT-Integration and created a new workflow. The workflow description information in the sample material was “serum spectrum collection.” The spectrum collection method in “sample specification” was the transmission method (transmission module). The number of scans set in “Number of Scans” was 64. In the “Resolution” option, we made sure that the resolution of the collected spectrum was 8 cm^−1^. In the “Data format” option, we confirmed that the absorbance was the spectral data format. We kept the sample in the refrigerator at −80°C. Considering that room temperature andrelative humidity may affect serum status, we control the room temperature at24°C and humidity around 44%. In this environment, we took out the samples tobe tested from the refrigerator and equilibrated at room temperature (2h). Wethen turned on the Antaris II Fourier transform near-infrared spectrometer andwarmed up the instrument (0.5h). After preheating, we accurately pipet 300ul ofserum samples into a cuvette with a 5mm optical path length. After that, weopen the sample cell of the instrument and put the cuvette with the sample intothe sample cell. In order to eliminate the influence of the background, water blank control calibration was collected every hour.

### 2.4. Sample Preparation and Reference Analysis of HPLC

HPLC reference analysis was performed immediately after the near-infrared spectrum of the sample was collected. We aspirated 200 ul of the serum sample in a 1.5 ml centrifuge tube, accurately drew 30 ul of 0.72 mg/mL *γ*-aminobutyric acid stock solution, and added it to a 1.5 ml centrifuge tube as an internal control; then, we added 600 ul of 3% sulfosalicylic acid solution to precipitate the protein centrifuged at 12000 r/min for 20 minutes, took the supernatant for use. Derivatization treatment: Precisely pipette 200ul of the above supernatant into a new 1.5ml centrifuge tube, pipette 20ul of 0.63 mg/mL theanine stock solution as an internal reference, add 120ul of acetonitrile solution, 20ul of triethylamine solution, and the concentration of 100ul is 0.2 mol/l phenyl isothiocyanate acetonitrile solution, mix well in a vortex shaker for 30s, seal with a parafilm, derivatize in a constant temperature water bath at 40°C for 1 hour, take out and add 400ul of n-hexane, and mix in a vortex shaker for 60s After standing for 10min. The supernatant was taken into the sample. We chose a Hypersil C18 column (250 mm × 4.6 mm, 5 *μ*m), and the injection volume per needle was 4 ul, the constant flow rate was 1 ml/min, the constant column temperature was 35°C, and the detection wavelength was 254 nm; the mobile phase A is ammonium acetate solution (PH=6.5, concentration=0.05mol/l), and mobile phase B is acetonitrile.. The time nonconcentration gradient was as follows: 0 min, 0% B; 0∼13 min, 0∼5% B; 13∼16 min, 5∼8% B; 16∼16.1 min, 8∼2% B; 16.1∼30 min, 2% B; 30∼32 min, 2∼7% B; 32∼40 min, 7∼17% B; 40∼54 min, 17∼25% B; 54∼58 min, 25∼30% B; and 58∼65 min, 30∼60% B. The above results were used as reference results for near-infrared analysis. We took the corresponding volume of each standard stock solution, added 0.1 mol/l hydrochloric acid to make the volume to 10 ml, and mixed it well. The pretreatment method was the same.

### 2.5. Spectral Data Analysis and Removing Outlier Samples

TQ Analyst 9 (Thermo Fisher Scientific, Waltham, MA, USA) was used for NIR spectrometer control and data analysis. We opened the original spectrum data in OMNIC and saved the Matlab identifiable data. We imported the CSV text file into MATLAB to save the work area. In the quantitative method, after determining the concentration information of each component and the standard spectrum or characteristic spectrum range, the Mahalanobis distance was calculated on this basis. Each standard spectrum was ranked according to its distance from the mean, Dixon' test and Chauvenet's criterion were used to test whether the outlier difference was significant, and the Liqun spectrum was eliminated.

### 2.6. Chemometric Methods

#### 2.6.1. iPLS Variable Selection

The principle of iPLS was to divide the entire spectral region into several subintervals, establish a partial least square regression model in the spectral region and each subinterval, and compare the accuracy of each model. The subinterval of the model with the highest accuracy value was the wavenumber range with the highest correlation with the target component. Simulation modeling is based on the iPLS algorithm. Optimal spectral range was calculated in MATLAB. We compared the effects of full-spectrum modeling and iPLS modeling.

#### 2.6.2. Simulated Annealing to Select Characteristic Variables

A vector V with a length of 1557 was used to store the retention of the feature, and each feature was coded, 0 means removing the feature and 1 means retaining the feature; the number of retained features was m. The optimization of this problem was actually to make 1 in the feature retention vector V as few as possible (reduce the number of features m), and the correlation coefficient calculated according to the retained features was as large as possible.

**Figure 1:**
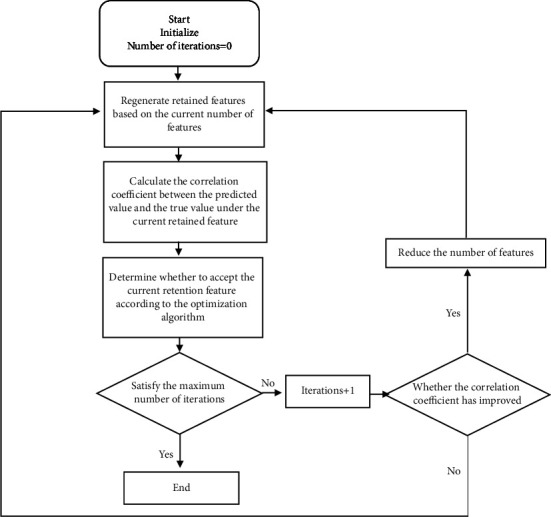
Algorithm design diagram.

Therefore, the designed algorithm was as follows:

  Step 1. We generated the initial feature retention vector V, and the number of features *m* was less than 1557. Partial least squares regression was performed based on the current retained features, and the correlation coefficient between the predicted value and the true value under the test set was calculated.
  Step 2. We rearranged the feature retention vector V according to the current feature number m and changed the distribution of retained features.
  Step 3. We performed partial least squares regression based on the current retained features and calculated the correlation coefficient between the predicted value and the true value under the test set.
  Step 4. We determined whether to accept the new feature retention vector V according to the optimization algorithm.
  Step 5. We determined whether the maximum number of iterations was met, and if so, exited directly.
  Step 6. We determined whether the correlation coefficient was improved compared to the global value under the new feature retention vector V. If there was an improvement, we reduced the number of features m and then executed Step 2; if there was no improvement, we returned to Step 2.
  The algorithm design steps are shown in Figure 1.

The simulated annealing algorithm was used to determine whether to accept the new solution, in order to prevent the situation from being limited to the local optimum. The core of simulated annealing lies in the Metropolis criterion:

(1) $$\begin{array}{c} P=\left\{\begin{array}{cc} 1, & \Delta E\leq 0,\\ e^{-\Delta E/T}, & \Delta E>0. \end{array}\right. \end{array}$$

In the formula, *P* represents the probability of accepting a new solution, Δ*E* is the function change value (in this calculation, it represents the difference between the previous correlation coefficient and the next), and *T* is the temperature. Δ*E* ≤ 0 means that if the solution becomes better, the new solution must be accepted; Δ*E* > 0 indicates if the solution becomes worse, the new solution will be accepted according to a certain probability; that is, it will jump out of the local optimum.

### 2.7. Metrics for Evaluation

After the establishment of the quantitative model, its performance needs to be evaluated. The main inspection indicators were the mean square error (RMSEC), correlation coefficient (*R*), and root mean square error of cross validation (RMSECV). The main calculation formulas are as follows:

(2) $$\begin{array}{c} \text{RMSEC}=\sqrt{\frac{\sum \;\left({\hat{C}}_{i}-C_{i}\right)}{n}},\\ R=\sqrt{1-\frac{\sum {\left({\hat{C}}_{i}-C_{i}\right)}^{2}}{\sum {\left(C_{i}-\bar{C}\right)}^{2}}}. \end{array}$$

*C*_*i*_ is the value measured by the standard chemical method, ${\hat{C}}_{i}$ is the value calculated by the near-infrared method, $\bar{C}$ is the average value, and *n* is the number of samples in the calibration set. The closer the *R* value is to 1 and the smaller the RMSEC, the better the stability of the established model and the stronger the predictive ability.

## 3. Results and Discussion

### 3.1. Proline Content in Serum

**Figure 2:**
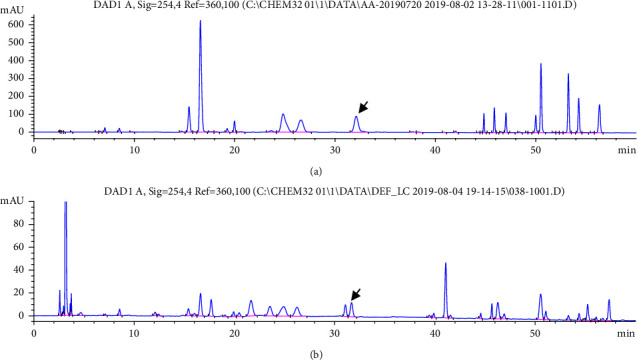
The HPLC chromatogram of amino acid, the arrow indicates proline, and amino acid mixed standard solution (a) and the serum sample (b).

All collected samples were analyzed using the high-performance liquid chromatography (HPLC) method described in Materials Method 4. A representative serum chromatogram is shown in Figure 2, which reflects that main amino acids in the serum were all baseline separated. Through HPLC experiments, it was found that the proline content in the serum was 0.005234 ± 0.001866 mg/ml (mean ± SD).The regression curve was *Y* = 0.3752*x*-14.28, *R*2 = 0.9979. The highest proline content in the sample was 0.008198 mg/ml, and the lowest proline content was 0.001917 mg/ml. The sample was randomly divided into two groups: calibration set and validation set. The former was used for modeling and the latter was used to test the accuracy of the model. Using the Kennard–Stone (*K*–*S*) algorithm, by maximizing the Euclidean distance between the selected object and the remaining objects, the ratio between the two sets was even. The mean values of the two groups were 0.005296 ± 0.001897 mg/ml and 0.005213 ± 0.001832 mg/ml, and the coefficients of variation were 0.3581 and 0.3514, respectively. There was no statistical difference between the two groups of means, *P* > 0.05. The random grouping result can be used in subsequent experiments. The results are shown in Table 1.

### 3.2. Near-Infrared Spectroscopy Basic Characteristics

**Figure 3:**
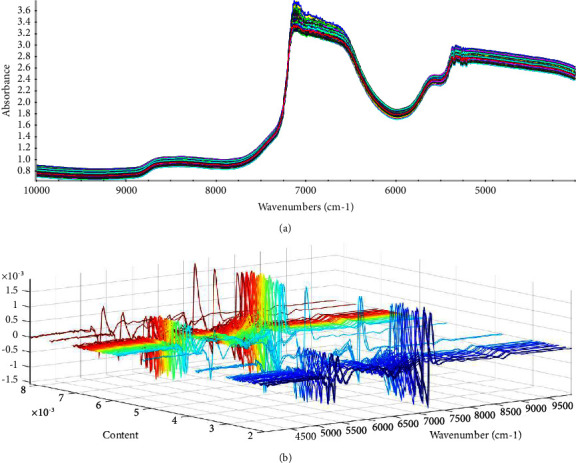
Serum NIR. (a) Original spectrum. (b) Second derivative/Norris derivative.

Based on the determination of serum proline, there were a total of 207 spectral samples in this analysis.  Figure 3(a) shows the original near-infrared spectra of the collected samples. The spectral uniformity was good, which better reflected the physical and chemical properties of the serum. The NIR spectra features with the overtones and combinations of species contain H groups such as -OH, -CH, and -NH. The water content of serum accounts for about 90%, and the H-OH structure has strong absorption in the entire infrared spectrum [36]. The solvation of water and the change of cluster structure had a great influence on the structure of water. Therefore, the near-infrared spectrum of the solvent water in the solution also contains a large amount of information about the solute [37]. By measuring the serum spectrum, it is theoretically possible to analyze serum proline, but data processing methods such as multivariate analysis are required to calculate the information of a single molecule or structure. It is known that the frequency-doubled spectrum often contains the interference of some overlapping peaks, and original NIR cannot directly show the absorption peak of a single substance or structure. Preprocessing the original spectrum, improving the signal-to-noise ratio, and removing invalid mutations are necessary steps to establish a high-performance model. In order to eliminate problems such as baseline drift and scattering effects, the second derivative (SD) can be a better choice. On this basis, an image denoising method based on the Norris derivative filtering algorithm is proposed. Norris noise filtering can effectively remove the noise increase caused by the derivative. Spectral preprocessing uses Second derivative/Norris derivative (5th degree polynomial, 5 point window). The spectrum processed by SD/Norris is shown in Figure 3(b), where each spectrum contains 1557 points, that is, 1557 features.

### 3.3. Comparison of Spectral Preprocessing Results

In order to solve the spectral drift or shift that appears in the process of spectral measurement, derivative processing is one of the important methods to purify the spectrum, usually by first-order or second-order differential processing. Derivative processing can also play a vital role in amplifying and separating overlapping information. However, it is important to note that the noise signal will also be amplified when the spectrum is differentiated. In order to avoid introducing new interference, it is necessary to smooth the spectrum to improve the signal-to-noise ratio and reduce random noise, thereby improving the stability of the model. There are two commonly used smoothing methods, one is the classic Savitzky–Golay filter and the other is the Norris derivative filter. Selection of the derivative and smoothing were usually carried out as needed. We evaluated the impact of 5 different spectrum pre-processing methods on the accuracy of the full-spectrum PLSR model verified by 207 repeated independent models. The detailed results of the comparison of each treatment method are shown in Table 2. We synthesize the modeling parameters for each treatment method. We determine that the derivative processing is the second derivative, and the smoothing processing is Norris-Derivative filtering. On this basis, the Rp of our established model is 0.77, which is superior to other processing methods.

### 3.4. Stepwise Multiple Linear Regression (SMLR) Result

**Figure 4:**
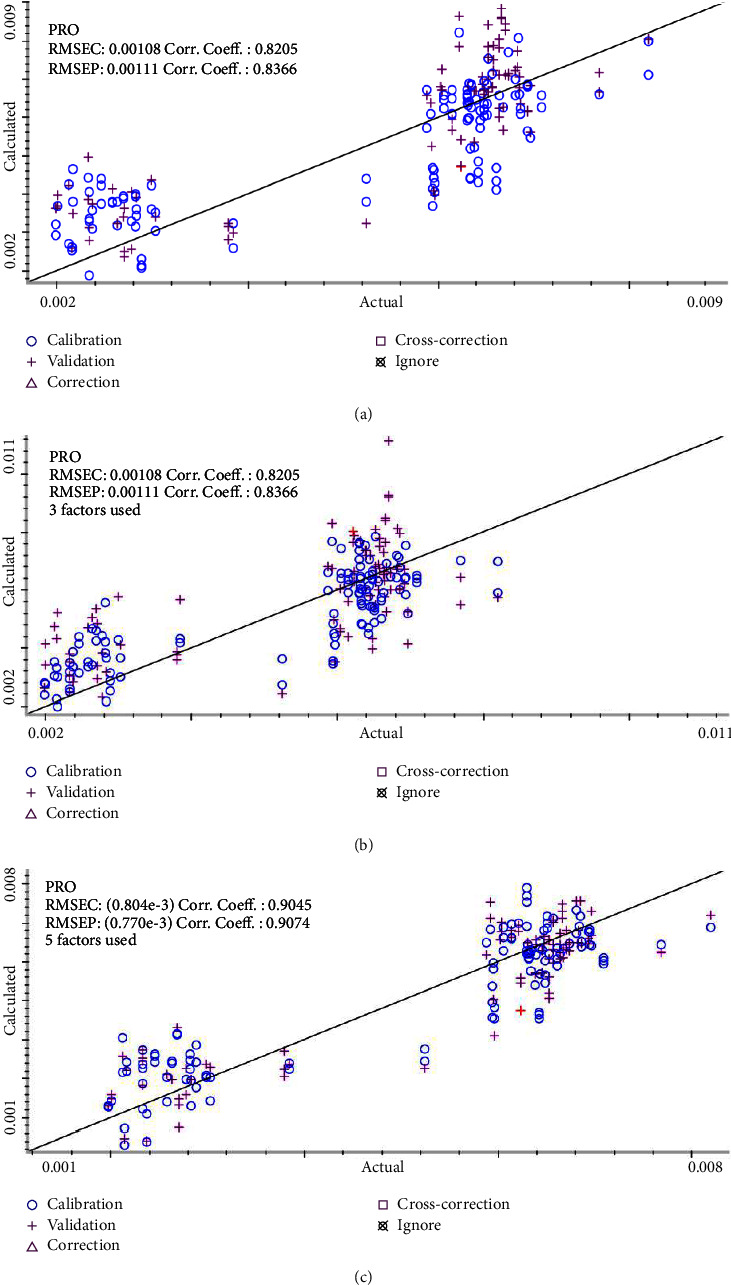
Prediction results of the (a) SMLR models, (b) PLS models, and (c) iPLS models.

SMLR is a more commonly used method in NIR. After each new independent variable is introduced forward, the substituted independent variable must be recalculated to check whether it continues to remain in the equation. We value and use this as a basis for the introduction and removal of independent variables alternately until no new variables are introduced or removed. Based on this principle, the wavenumber was selected as (9503.48–7347.46 cm^−1^), and the modeling results were as follows. It can be seen from Figure 4(c) that the performance of the SMLR model was general and cannot meet the experimental expectations because it may lose some important spectrum information, thereby reducing the predictive ability of the model.

### 3.5. PLS Results

PLS interacts with matrix factorization and regression, so the eigenvectors are directly related to the attributes of the sample. At the same time, it compensates for the interference caused by light scattering and other components, making the model more robust and suitable for complex component systems, such as multiple analysis samples of mixed solutions and biological fluids. Based on the full-spectrum PLR modeling results shown in Figure 4(b), the results of the calibration set and validation set of the PLS model were both poor, and both were worse than those of the SMLR model, which indicated that full-spectrum (4000–10000 cm^−1^) information was redundant; the variables needed to be further streamlined and the model optimized.

### 3.6. iPLS Modeling Results

The model is built using traditional modeling methods, but the overall performance of the built model did not lead to a satisfactory effect. In order to optimize the model, using the idea of the iPLS algorithm, the full spectrum is equally divided into N subintervals, and PLS models are established in different wavenumber ranges. Comparing different division methods and different characteristic wavenumber ranges, it is finally determined that full spectrum was divided into nine segments, and the wavenumber range was selected as (7335.89–7999.28 cm^−1^) to establish the model with the best effect shown in Figure 4(b). This is different from the characteristic range of 7352 cm^−1^, 8620 cm^−1^, and 5988 cm^−1^ in proline solution determined by Tao et al. [38]. This may be different from the stretching and deformation of the CH, COOH, and NH structure in the proline structure. It is caused by the mutual interference of other substances in serum. In fact, the idea of the iPLS algorithm is to equally divide the entire spectrum mechanically, which may have major risks such as fragmentation or loss of characteristic signals, and it is impossible to extract theoretically complete and effective information. However, it should be noted that the spectra of the serum samples are special, and there are a large number of other solute effects, which are quite different from the proline standard solution, and further analysis is required.

### 3.7. Simulated Annealing (SA) Modeling Result

**Figure 5:**
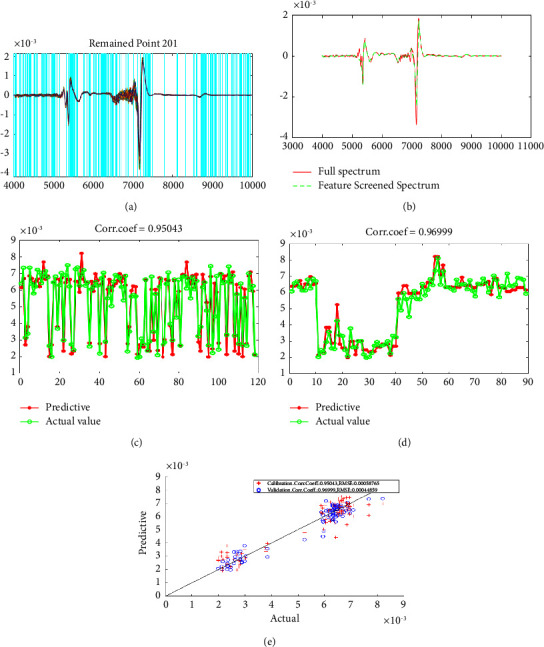
(a) The distribution of the retained features. (b) The comparison of the reconstructed spectrum after screening the features with the original full spectrum. (c) The comparison between the predicted value of the verification set and the actual value. (d) The comparison between the predicted value of the calibration set and the actual value. (e) Final model effect.

There are too many features in full spectrum. When performing regression, noncritical features can be omitted, which not only improves the calculation efficiency but also improves the accuracy of the regression. The simulated annealing algorithm is used to screen 1557 features of full spectrum. The essence of the algorithm is to address the problem of sufficient feature information extraction. We know that the spectral curves obtained in the experiment are composed of 1577 discrete points. Thus, the problem is transformed into screening 1557 variables of the full spectrum, keeping or removing each variable, as shown in Figures 5(a) and 5(b). In the end, we build the model based on keeping all the variables and calculate the relevant parameters so that the model can have the best effect. In the final result, the number of retained feature variables was 201, and the correlation coefficient of the built model increased to 0.9700, as shown in Figures 4(c)–4(e). This result had obvious advantages over traditional modeling methods [39–41]. At the same time, the effect of this model was also better than that of other models in this study. The detailed comparison results are shown in Table 3. However, some facts needed to be recognized that the object of this study was the NIR characteristics of proline in serum, not the NIR characteristics of a single proline solution, which may have certain reference value for the development of clinical serum spectroscopy applications.

## 4. Conclusion

Pro plays different roles during different biological processes, affecting the biological processes in a living cell [12–14, 19]. For example, proline metabolism involves the interconversion of proline and glutamate, which via the sequential action of proline dehydrogenase (ProDH) and P5C dehydrogenase converts proline successively to P5C then to glutamate in mitochondria [10]. This is a process that is directly linked to cellular energetics through the respiratory electron transport chain, which is an important part of regulating the redox equilibrium reaction. Overall, proline metabolism plays an important role in the process of occurrence and development of diseases. Thus, monitoring the changes in the concentration of proline under physiological or pathological conditions is significant for disease prevention and management in the future.

In this study, we have developed a nondisruptive and convenient method for rapid detection of proline in serum. First, we took a traditional approach to developing models using TQ Analyst 9 (Thermo Fisher Scientific, Waltham, MA, USA). Preliminary experiments indicated that the model directly established with raw spectral data as a variable has poor effects. Due to this, various preprocessed algorithms were used to deal with the raw spectral data to ensure the high accuracy and precision of quantitative models. From the perspective of spectral pretreatment methods, the soil NIR spectra processed with different pretreatment methods showed different modeling effects. The calculation result showed that the Norris derivative filter was better than the Savitzky–Golay filter, while the second derivative of the raw spectral data was better than the first derivative. In overall consideration, we choose the raw spectrum preprocessed method as the second derivative spectrum + Norris derivative filter. Finally, by comparing the four algorithms of SMLR, PLS, iPLS, and simulated annealing, further optimization of the model is completed. All in all, the final development of this research is based on serum near-infrared spectroscopy to establish and optimize the proline detection method, in which the simulated annealing model is better than the traditional near-infrared model. In addition, compared with traditional high-performance liquid chromatography detection methods, this nondestructive and rapid detection method has obvious advantages, or it can achieve rapid nondestructive detection of serum proline, providing new ideas for the development of new detection methods in the medical field. However, this requires more samples to confirm these results and develop more robust detection models in further research.

## Figures and Tables

**Table 1:** Serum proline content and its grouping situation.

	Statistics	Number	Max (mg/ml)	Min (mg/ml)	Mean ± SD (mg/ml)	CV
PRO	Calibration set	118	0.008198	0.002004	0.005296 ± 0.001897	0.3581
	Validation set	89	0.007682	0.001982	0.005213 ± 0.001832	0.3514

**Table 2:** Comparison results of preprocessing methods.

	Rc	Rp	SEC	SEP	Offset	RPD	RMSEC	RMSEP
Raw spectrum	0.40	0.27	0.00007	0.00011	−0.0459	14.39	0.00173	0.00183
1D + SG	0.51	0.64	0.00009	0.00013	0.0210	14.34	0.00162	0.00139
1D + NO	0.74	0.74	0.00015	0.00013	−0.0209	14.51	0.00126	0.00124
2D + SG	0.57	0.71	0.00011	0.00011	0.0227	14.34	0.00155	0.00130
2D + NO	0.74	0.77	0.00013	0.00018	−0.0013	14.49	0.00126	0.00125

Note: SEC, calibration set standard error; SEP, prediction set standard error; RPD, relative percentage difference.

**Table 3:** Different algorithm optimization results.

	Calibration set		Validation set		LVs
	Rc	RMSEC	Rp	RMSEP	
SMLR	0.8205	0.00108	0.8366	0.00111	
PLS	0.8190	0.00108	0.6744	0.00150	4
IPLS	0.9045	0.000804	0.9074	0.000770	5
SA	0.9504	0.000588	0.9700	0.000449	5

## Data Availability

The data used to support the results of this study are consistent with the data in this paper. Any further information and algorithm code are available from the corresponding authors upon request.

## References

[1] Barrett G. (2012). *Chemistry and Biochemistry of the Amino Acids*.

[2] Nie C., He T., Zhang W., Zhang G., Ma X. (2018). Branched chain amino acids: beyond nutrition metabolism. *International Journal of Molecular Sciences*.

[3] Lieu E. L., Nguyen T., Rhyne S., Kim J. (2020). Amino acids in cancer. *Experimental and Molecular Medicine*.

[4] Kelly B., Pearce E. L. (2020). Amino Assets: How Amino Acids Support Immunity. *Cell Metabolism*.

[5] Wang W., Zou W. (2020). Amino acids and their transporters in T cell immunity and cancer therapy. *Molecular Cell*.

[6] Hu C. A., Delauney A. J., Verma D. P. (1992). A bifunctional enzyme (delta 1-pyrroline-5-carboxylate synthetase) catalyzes the first two steps in proline biosynthesis in plants. *Proceedings of the National Academy of Sciences*.

[7] Savouré A., Jaoua S., Hua X. J., Ardiles W., Van Montagu M., Verbruggen N. (1995). Isolation, characterization, and chromosomal location of a gene encoding the Δ^1^-pyrroline-5-carboxylate synthetase in *Arabidopsis thaliana*. *FEBS Letters*.

[8] Szoke A., Miao G.-H., Hong Z., Verma D. P. S. (1992). Subcellular location of *δ*1-pyrroline-5-carboxylate reductase in root/nodule and leaf of soybean. *Plant Physiology*.

[9] Verbruggen N., Villarroel R., Van Montagu M. (1993). Osmoregulation of a pyrroline-5-carboxylate reductase gene in *Arabidopsis thaliana*. *Plant Physiology*.

[10] Adams E. (1970). Metabolism of proline and of hydroxyproline. *International Review of Connective Tissue Research*.

[11] Donald S. P., Sun X. Y., Hu C. A. (2001). Proline oxidase, encoded by p53-induced gene-6, catalyzes the generation of proline-dependent reactive oxygen species. *Cancer Research*.

[12] Natarajan S. K., Zhu W., Liang X. (2012). Proline dehydrogenase is essential for proline protection against hydrogen peroxide-induced cell death. *Free Radical Biology and Medicine*.

[13] Liu W., Glunde K., Bhujwalla Z. M., Raman V., Sharma A., Phang J. M. (2012). Proline oxidase promotes tumor cell survival in hypoxic tumor microenvironments. *Cancer Research*.

[14] Liu W., Le A., Hancock C. (2012). Reprogramming of proline and glutamine metabolism contributes to the proliferative and metabolic responses regulated by oncogenic transcription factor c-MYC. *Proceedings of the National Academy of Sciences*.

[15] Miller G., Honig A., Stein H., Suzuki N., Mittler R., Zilberstein A. (2009). Unraveling Δ^1^-pyrroline-5-carboxylate-proline cycle in plants by uncoupled expression of proline oxidation enzymes. *Journal of Biological Chemistry*.

[16] Phang J. M. (1985). The regulatory functions of proline and pyrroline-5-carboxylic acid. *Current Topics in Cellular Regulation*.

[17] Washington J. M., Rathjen J., Felquer F. (2010). L-Proline induces differentiation of ES cells: a novel role for an amino acid in the regulation of pluripotent cells in culture. *American Journal of Physiology—Cell Physiology*.

[18] Phang J. M., Liu W., Hancock C., Christian K. J. (2012). The proline regulatory axis and cancer. *Frontiers in Oncology*.

[19] Zarse K., Schmeisser S., Groth M. (2012). Impaired insulin/IGF1 signaling extends life span by promoting mitochondrial L-proline catabolism to induce a transient ROS signal. *Cell Metabolism*.

[20] Ijarotimi O. S., Olopade A. J. (2009). Determination of amino acid content and protein quality of complementary food produced from locally available food materials in ondo state, Nigeria. *Malaysian Journal of Nutrition*.

[21] Song Y., Xu C., Kuroki H., Liao Y., Tsunoda M. (2018). Recent trends in analytical methods for the determination of amino acids in biological samples. *Journal of Pharmaceutical and Biomedical Analysis*.

[22] Petritis K., Elfakir C., Dreux M. (2002). A comparative study of commercial liquid chromatographic detectors for the analysis of underivatized amino acids. *Journal of Chromatography A*.

[23] Eggum B. O., Sørensen H. (2018). *Absorption and Utilization of Amino Acids*.

[24] Nemkov T., D’Alessandro A., Hansen K. C. (2015). Three-minute method for amino acid analysis by UHPLC and high-resolution quadrupole orbitrap mass spectrometry. *Amino Acids*.

[25] Zhang B., Rong Z., Shi Y., Wu J., Shi C. (2011). Prediction of the amino acid composition in brown rice using different sample status by near-infrared reflectance spectroscopy. *Food Chemistry*.

[26] Fan W.-P., Wu J. (2007). Determination of amino acid content in products by spectrophotometric method. *China Measurement Technology*.

[27] Liu Z., Liu B., Pan T., Yang J. (2013). Determination of amino acid nitrogen in tuber mustard using near-infrared spectroscopy with waveband selection stability. *Spectrochimica Acta Part A: Molecular and Biomolecular Spectroscopy*.

[28] Liu X., Zhang X., Rong Y. Z., Wu J. H., Yang Y. J., Wang Z. W. (2015). Rapid determination of fat, protein and amino acid content in coix seed using near-infrared spectroscopy technique. *Food Analytical Methods*.

[29] Ouyang Q., Chen Q., Zhao J., Lin H. (2013). Determination of amino acid nitrogen in soy sauce using near infrared spectroscopy combined with characteristic variables selection and extreme learning machine. *Food and Bioprocess Technology*.

[30] Kovalenko I. V., Rippke G. R., Hurburgh C. R. (2006). Determination of amino acid composition of soybeans (glycine max) by near-infrared spectroscopy. *Journal of Agricultural and Food Chemistry*.

[31] Beć K. B., Grabska J., Huck C. W. (2020). Near-infrared spectroscopy in bio-applications. *Molecules*.

[32] Wang Y., Deng G., Xu J., Wang Z., Gu Q., Zeng J. (2018). Rapid detection method for the content of insensitive agent in single-base propellant. *Journal of Gun and Explosive Medicine*.

[33] Chu X., Shi Y., Chen P., Li J., Xu Y. (2019). Progress in the research and application of near-infrared spectroscopy analysis technology in my country in the past five years. *Chinese Journal of Instrumental Analysis*.

[34] Li J., Zhou Z., Huang S. (2018). The application of near-infrared spectroscopy to the chemometric modeling of online quality control of Chinese herbal medicine oral liquid. *Progress in Chemical Industry*.

[35] Zhu X., Cui X., Cai W., Shao X. (2018). Temperature-controlled near-infrared spectroscopy for the study of hydrogen bond interaction of amine compounds. *Acta Chimica Sinica*.

[36] Ron-Harel N., Ghergurovich J. M., Notarangelo G. (2019). T cell activation depends on extracellular alanine. *Cell Reports*.

[37] Fan M., Zhao Y., Liu Y., Cai W., Shao X. (2015). Near infrared spectroscopy water spectromics. *Progress in Chemistry*.

[38] Tao L., Huang W., Yang X. (2016). The correlation between 20 amino acids near infrared spectra and their molecular structures. *Spectroscopy and Spectral Analysis*.

[39] Xie C., Xu N., Shao Y., He Y. (2015). Using FT-NIR spectroscopy technique to determine arginine content in fermented cordyceps sinensis mycelium. *Spectrochimica Acta Part A: Molecular and Biomolecular Spectroscopy*.

[40] Shen F., Niu X., Yang D. (2010). Determination of amino acids in Chinese rice wine by fourier transform near-infrared spectroscopy. *Journal of Agricultural and Food Chemistry*.

[41] Belščak-Cvitanović A., Valinger D., Benkovic M. (2017). Integrated approach for bioactive quality evaluation of medicinal plant extracts using HPLC-DAD, spectrophotometric, near infrared spectroscopy and chemometric techniques. *International Journal of Food Properties*.

